# Independent Living for Older Adults with Cognitive Impairment: A Narrative Review of Stakeholder Perceptions and Experiences with Assistive and Socially Assistive Robots

**DOI:** 10.3390/jal5030034

**Published:** 2025-09-15

**Authors:** Delaram Sirizi, Morteza Sabet, Katelyn Hummel, Juanita-Dawne R. Bacsu, Ava Longo, Zahra Rahemi

**Affiliations:** 1 Department of Biological, Environmental, and Earth Sciences, University of South Carolina Aiken, Aiken, SC 29801, USA; 2 School of Mechanical and Automative Engineering, Clemson University, Greenville, SC 29607, USA; 3 School of Nursing, Clemson University, Clemson, SC 29634, USA; 4 School of Nursing, Thompson Rivers University, Kamloops, BC V2C 0C8, Canada

**Keywords:** Alzheimer’s disease, dementia, cognitive impairment, human–robot interactions, artificial intelligence, independent living, robot-assisted support

## Abstract

**Background:**

Alzheimer’s disease and related dementias (ADRD) are a major cause of mortality among older adults globally. The cognitive decline associated with ADRD often reduces individuals’ ability to live independently over time, increasing reliance on caregivers. Assistive and socially assistive robots offer a promising means of supporting independent living. This narrative review examined how older adults with ADRD, their caregivers, and healthcare providers perceive and experience interactions with robots.

**Methods:**

Guided by the Population, Phenomenon of Interest, and Context (PICo) framework, five databases were searched. Sixteen studies met the inclusion criteria. Extracted data were summarized, and a convergent synthesis integrated qualitative and quantitative findings.

**Results:**

Drawing on content analysis, the qualitative findings were organized into three domains: user perceptions and experiences, barriers to adoption, and suggestions for improvement. Quantitative results emphasized usability, usefulness, acceptance, satisfaction, feature preferences, and barriers. While most stakeholders viewed robots as beneficial, acceptance was shaped by factors such as design features, timing of introduction, familiarity with technology, and perceived need.

**Conclusions:**

This review highlights priorities for future research and development, including personalization, ethical safeguards, and caregiver integration, to improve the acceptance and effectiveness of robot-assisted support for individuals with cognitive impairment.

## 1. Introduction

Alzheimer’s disease and related dementias (ADRD) represent a major global public health challenge. Worldwide, ADRD are the seventh leading cause of death among older adults, with an estimated 57 million people living with dementia in 2021 and nearly 10 million new cases diagnosed each year [1]. This burden is projected to increase substantially as populations age across regions. For example, in the United States (U.S.), ADRD ranked as the fifth leading cause of death among adults aged 65 and older currently, affecting 6.9 a number projected to reach 14 million by 2060 [2]. Cognitive decline in ADRD progressively impairs individuals’ ability to live independently, increasing reliance on others over time [1,3]. As the population of older adults grows, enabling independent living and improving quality of life have become priorities for individuals, healthcare systems, and policymakers [4]. This requires a holistic approach that values innovation alongside individuals’ needs and values, as well as broader environmental, economic, and systemic factors influencing care. Many older adults prefer to maintain their independence and remain at home [5]. While supporting aging in place can help lower healthcare costs by reducing hospitalization rates [6], it may shift rather than reduce caregiver workload, particularly increasing the demands placed on informal or family care partners. The “care-gap” refers to the difference between the functional abilities needed for independent living and those an individual actually possesses, a gap typically addressed by human caregivers [7]. Addressing this gap is critical in care planning as it directly affects caregiver workload and the sustainability of care.

A potential solution to reduce the growing workload of formal and informal caregivers of individuals with cognitive impairment is the use of technologies to assist with daily living [7]. This includes the use of robots and health-monitoring devices powered by advanced energy storage technologies [8–10]. Robotics has been applied in the care of individuals with mild cognitive impairment (MCI), Alzheimer’s disease, and related dementias, supporting therapy, cognitive stimulation, emotional well-being, and other activities [11]. The advancement of robotics offers promising avenues to support activities of daily living and improve independent living for individuals with cognitive impairment [12]. Evidence suggests that robots are generally well received by individuals with cognitive impairment and their caregivers, particularly when they provide practical and effective support [13].

Different types of robots offer varying services, including companionship and social interaction, assistance with daily living activities, cognitive engagement, and support for medication compliance and healthy diets [14]. These robots are categorized into two main types: (i) assistive robots, and (ii) social or socially assistive robots (SARs). Both assistive robots and SARs are designed to reduce loneliness, enhance mental health, and promote independence. Various types of assistive robots have been introduced to the market, including service robots, social robots, and companion robots, each created with different sizes, designs, and functionalities. Service robots can assist with daily routines and activities of daily living, enhance quality of life, and reduce caregivers’ workload [12]. In contrast, SARs, equipped with sensors, cameras, and microphones to detect and respond to human movements and emotions, provide emotional support and companionship. Companion robots, pet robots, cognitive assistive robots, and telepresence robots fall into this category. Although their primary functions differ, service and social assistive robots share overlapping features, particularly in supporting cognitive engagement. Cognitive stimulation is critical for individuals with cognitive impairment, helping maintain mental acuity and improving independence through physical assistance with everyday tasks [15].

The successful implementation of robots for individuals with cognitive impairment depends heavily on user acceptance. Studies have evaluated the effectiveness of robots among older adults with ADRD, but to our knowledge, few have explored the perspectives of different care stakeholders as end users of this technology. Understanding these perspectives is essential to improving the design and acceptance of assistive robots, thereby enhancing their usability among individuals with cognitive impairment and their caregivers.

In our previous work, we reviewed human–robot interactions among older adults, identifying eight key factors influencing robot acceptance, including usefulness, ease of use, safety, and emotional connection [16]. However, that study focused broadly on older adults and included only qualitative studies. To build upon this work, the current narrative review synthesizes both qualitative and quantitative studies examining stakeholder perspectives, including those of older adults with cognitive impairment, their caregivers, and healthcare providers, regarding assistive and social robots designed to support independent living.

The specific aim of this review is to provide a comprehensive understanding of stakeholder perspectives on robot-assisted support for older adults with cognitive impairment. We aimed to address three questions: (1) What are the perceived benefits and challenges of assistive and social robots for older adults with cognitive impairment? (2) How do caregivers and healthcare providers view the role of robots in supporting independent living? (3) What ethical, emotional, and practical considerations influence stakeholder acceptance of robots in dementia care?

## 2. Materials and Methods

**Figure 1.**
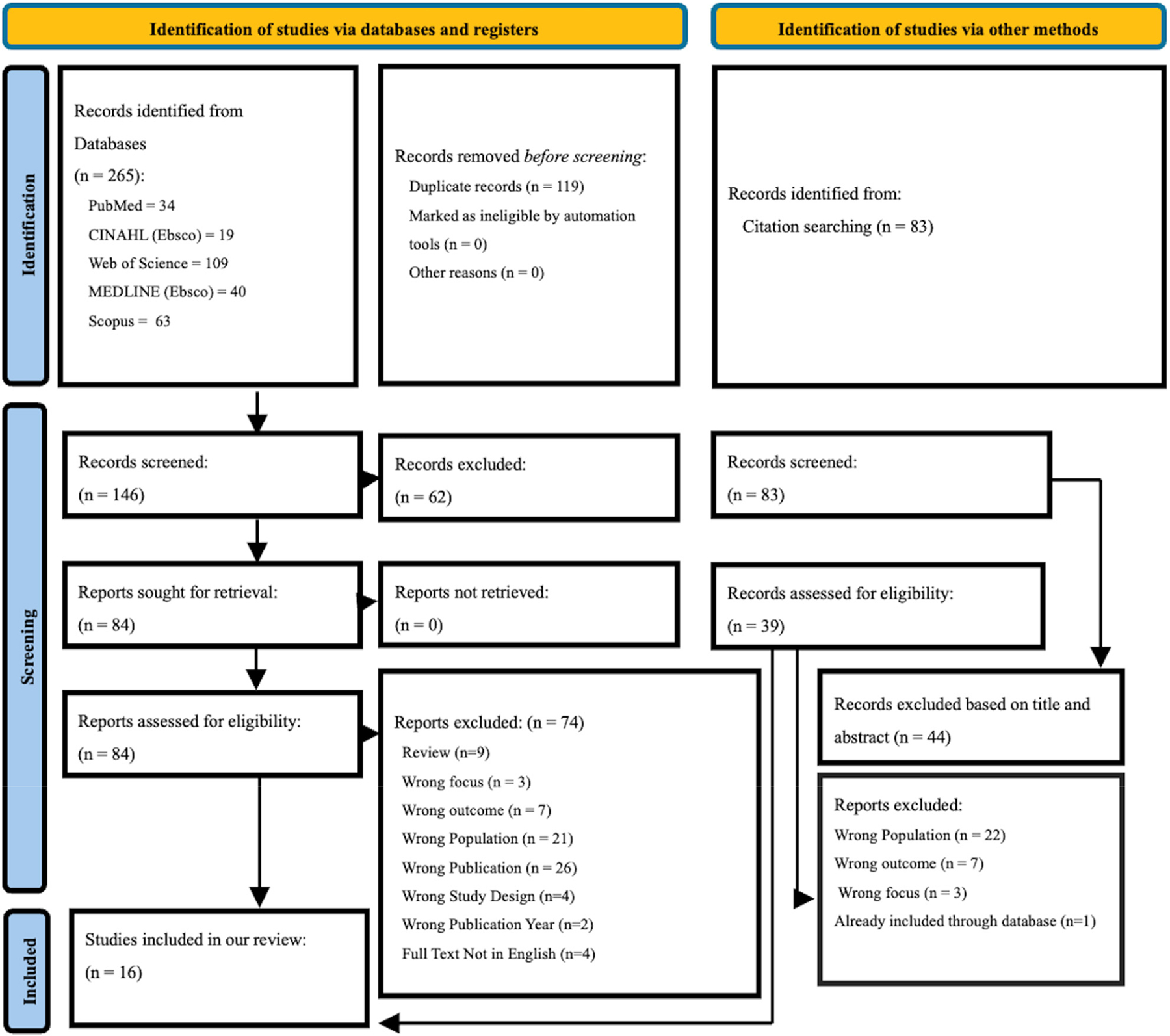
The search diagram illustrating the study selection process.

We conducted a narrative review, following systematic practices [17], to synthesize diverse evidence on stakeholder perspectives regarding assistive robots for individuals with cognitive impairment. This approach was chosen to accommodate the emerging, heterogeneous nature of the literature, which spans various study designs and populations. We used the Population, Phenomenon of Interest, and Context (PICo) framework to formulate the review’s aims and guide data collection, as it is well suited for studies exploring experiences and perceptions [18,19]. We used a structured search strategy, predefined inclusion and exclusion criteria, and included a flow diagram to document the study selection process (Figure 1). In May 2024, we searched five databases: PubMed, Web of Science (all collections), CINAHL (Ebsco), MEDLINE (Ebsco), and Scopus. Search strings were developed using Medical Subject Headings (MeSH) terms and keywords derived from the PICo framework (Table S1), with detailed search strings adapted from PubMed (Textbox S1). Reference lists of key articles were also screened to identify additional studies.

### 2.1. Inclusion and Exclusion Criteria

Studies were included if they met the following criteria: (a) published in English between 2019 and 2024, to capture the most recent developments in robotics and artificial intelligence (AI); (b) published in peer-reviewed journals with full-text available; and (c) focused on the experiences or perspectives of stakeholders regarding robots designed to enhance independent living. Stakeholders included older adults with Alzheimer’s disease, ADRD, or MCI living independently with or without caregiver support, as well as formal and informal caregivers and healthcare providers. Qualitative, quantitative, and mixed-methods studies were eligible for inclusion. Although the standard definition of “older adults” typically refers to individuals aged 65 and older, we expanded eligibility to include studies with participants aged 50 and older, aligning with research identifying middle-aged adults (50+) as a critical population for early detection, intervention, and the prevention of dementia-related decline [2]. Detailed inclusion and exclusion criteria are provided in Table S2.

### 2.2. Study Selection

The initial database search retrieved 265 studies, of which 118 duplicates were removed. Two independent reviewers (Anonymized-for-Review_1, Anonymized-for-Review_2) screened the remaining titles and abstracts using Covidence, a web-based tool for systematic review management [20]. Any disagreements between reviewers during the screening or full-text review stages were resolved through discussion and consensus. A third reviewer (Anonymized-for-Review_3) was consulted when agreement could not be reached. Following title and abstract screening, 84 studies were selected for full-text review. In addition, backward citation tracking of 83 references was conducted to identify further eligible studies. After full-text screening and consensus between the two reviewers, 16 studies met the inclusion and exclusion criteria and were included in the review. Figure 1 displays the study selection process. Detailed search queries for all databases are provided in Table S3.

### 2.3. Data Extraction

Two researchers (Anonymized-for-Review_1, Anonymized-for-Review_2) independently extracted, summarized, and recorded data from eligible studies using Covidence. A second review of the extracted data was conducted by two researchers (Anonymizedfor-Review_2, Anonymized-for-Review_3) to achieve consensus. Extracted data included: (1) study information (authors, publication year, country, robot type), (2) study purpose, (3) study methods (design, inclusion/exclusion criteria, data collection, data analysis), (4) participant characteristics (older adults with ADRD or MCI, caregivers, healthcare providers; number of participants, age, sex), (5) intervention or survey details (setting and duration), and (6) key findings (Table 1).

### 2.4. Data Synthesis

Studies were categorized as qualitative, quantitative, or mixed methods. As part of this narrative review, the methodological strengths of each study were critically reviewed to contextualize the findings. Findings within each methodological category were first synthesized independently. For mixed-methods studies, qualitative findings were integrated into qualitative synthesis (Section 3.1) and quantitative findings into quantitative synthesis (Section 3.2) to ensure consistent comparison across studies. We then integrated the results using a convergent synthesis approach, which involves comparing and combining qualitative and quantitative findings to produce a comprehensive understanding of the research topic while preserving the distinct contributions of each data type [35]. This convergent synthesis is described in the Discussion section.

## 3. Results

Sixteen peer-reviewed studies were included in the review, comprising qualitative (*n* = 8) [3,12,21,24,26–28,33], quantitative (*n* = 1) [34], and mixed-methods designs (*n* = 7) [22,23,25,29–32]. Of these, twelve studies focused on SARs, including pet-like robots (*n* = 3) [22,23,28], humanoid robots (*n* = 2) [29,32], a virtual companion robot (*n* = 1) [25], machine-like robots (*n* = 4) [21,26,27,34], and telepresence robots (*n* = 2) [3,24]. Four studies examined assistive or service robots [12,30,31,33].

The studies were conducted across nine countries, including the U.S., Belgium, Canada, Poland, Taiwan, the United Kingdom (U.K.), New Zealand, and Mexico. Notably, Yuan et al.’s U.S.-based study collected data from 13 countries, with 92.25% of responses originating from the U.S.; the remaining countries included China, Germany, Japan, Australia, Slovakia, Canada, the U.K., India, Saudi Arabia, Taiwan, Hong Kong, and unspecified European nations [32]. Similarly, Coşar et al.’s U.K.-based study gathered data from the U.K., Greece, and Poland [30]. These 16 studies included data from 284 older adults with MCI or ADRD, 320 formal and informal caregivers, 27 healthcare providers, and 1 developer. One study included 171 cognitively healthy older adults [22], and another included 26 younger adults [31]. These studies were retained because they also reported findings from older adults with cognitive impairment. Furthermore, two studies drew from the same research project but employed different data analysis methods and reported distinct findings [26,27].

Several studies had limited rigor due to reasons such as small sample size (e.g., ref. [3,29]) with 3 participants each, hypothetical or brief exposure to the robots (e.g., ref. [12,22,31]), or lack of clarity in analysis methods (e.g., ref. [30,34]). This review included these studies because they offered valuable insights into stakeholders’ perceptions and early-stage intervention development. However, their findings were interpreted cautiously in the synthesis.

### 3.1. Qualitative Findings

This section presents qualitative findings drawn from both qualitative studies and the qualitative components of mixed-methods studies included in this review, organized into three overarching domains: (1) user perceptions and experiences, (2) barriers to adoption, and (3) suggestions for improving robot design and functionality. These domains were identified using a conventional content analysis approach, which involved coding and quantifying recurring concepts across studies to derive thematic categories. Although these domains are presented separately, many themes overlapped across studies. Definitions and supporting quotations are provided in Supplementary Table S4.

#### 3.1.1. Domain 1: User Perceptions and Experiences

##### Theme 1: Perceived Usefulness and Benefits

Across the reviewed studies, both SARs and assistive robots were generally perceived as valuable tools for supporting independent living among older adults with cognitive impairment. Perceptions of usefulness and benefit were shaped by robot type, functionality, and stakeholder group, with users evaluating both what robots could do and how those functions improved quality of life.

Among SARs, companion and telepresence robots were frequently evaluated in terms of their capacity for social engagement and emotional support. In one study comparing three robotic pets (including JustoCat, AIBO, and MiRo), participants found the robots more enjoyable than strictly useful, though JustoCat was perceived as both more useful and more enjoyable due to its realistic appearance and behavior [22]. In a study of a telepresence robot, older adults with dementia prioritized features such as medication reminders, medication dispensing, and emergency call functions, while caregivers and healthcare providers valued communication features for remote monitoring [24]. Similarly, caregivers valued companion robots for their ability to support social engagement, particularly by facilitating conversations around repetitive storytelling, a common feature in dementia care [32].

Assistive robots were generally valued for task-oriented support. Voice-activated reminder systems and communication features were found helpful by older adults with dementia for maintaining routines and compensating for memory loss [33]. Users of another assistive robot valued its object mapping and activity monitoring capabilities, although technical limitations were noted [30].

Beyond specific functionalities, several studies highlighted broader outcomes associated with robot use. Emergency call systems, environmental monitoring, and reminders contributed to a strong sense of safety and reassurance among both older adults with cognitive impairment and their caregivers [21,22,25,30,32,33]. Household task assistance, such as object retrieval and hygiene support, was also valued, particularly when it promoted greater independence in daily living [22,27,31]. However, some studies warned against over-reliance on robots for these tasks, cautioning that excessive assistance might reduce users’ motivation to stay actively engaged [12].

Robots that supported cognitive and physical engagement through memory games, exercise prompts, or step-by-step task guidance were viewed positively across multiple studies [24,30,32,33]. These functions were seen as beneficial for maintaining mental acuity and daily functioning. For socially isolated individuals with dementia, telepresence and companion robots provided a valued source of interaction and were sometimes the primary means of communication during periods of limited in-person contact [24]. Several studies also reported neuropsychiatric benefits, including reduced agitation, anxiety, and behavioral symptoms among individuals with dementia following regular engagement with companion robots [21,22,28,29].

Health-related features further enhanced the perceived benefit of both assistive robots and SARs. Participants appreciated applications for tracking vital signs, handling emergency situations, offering dietary reminders, and monitoring air quality in the home environment [24–27,30,32,33]. During the COVID-19 pandemic and periods of workforce shortage, robots were regarded as especially useful for maintaining care continuity when human caregivers were unavailable, although participants continued to express a strong preference for human interaction whenever feasible [25,26].

Finally, several studies identified populations most likely to benefit from robotic assistance. These included individuals with cognitive impairment, socially isolated older adults, and residents of supportive living environments [22,23]. However, limitations were noted for users with significant hearing loss or limited communicative ability, who may face barriers to fully engaging with robotic systems [29].

##### Theme 2: Acceptance and Satisfaction

Acceptance and satisfaction with robots were shaped by design characteristics, functionality, and the dynamics between caregivers and care receivers. Across studies, robots with realistic and familiar appearances, such as pet-like companion robots, were associated with greater acceptance compared to mechanical or abstract designs [21,22,28,32]. Active engagement also contributed to positive perceptions. Robots that initiated interactions, displayed photos, or responded meaningfully to user behavior were preferred over passive devices, and were associated with higher satisfaction and emotional comfort [21,23,33]. Harris-Gersten et al. reported that both older adults with dementia and their caregivers experienced feelings of relaxation and companionship during interactions with SARs [23].

Stakeholder differences influenced acceptance patterns. Caregivers tended to show higher acceptance of telepresence robots, valuing features such as remote control, communication functions, and home monitoring [24]. For older adults with cognitive impairment, robots sometimes offered a trusted alternative to human caregivers, particularly when trust issues were present. Ease of use and a sense of safety were consistently associated with higher satisfaction. Participants appreciated features that simplified operation, such as large buttons, clear screens, and voice activation [30,31,33]. Systems that provided timely alerts about errors or environmental risks enhanced both perceived safety and overall acceptance [31].

##### Theme 3: Emotional Connection

Emotional responsiveness further reinforced acceptance. Companion robots were frequently described as alleviating loneliness, reducing anxiety, and fostering a sense of companionship among older adults with MCI and ADRD [21,22,25,26,28,30,33]. In some cases, regular interaction with the robot was associated with a reduction in agitation and improvements in emotional well-being. Yuan et al. further found that participants reported feeling more at ease and experiencing lower anxiety levels when robots were integrated into the home environment [32].

##### Theme 4: Control and Autonomy

Robots were perceived as having a dual role in shaping users’ sense of control and autonomy. Several studies suggested that robotic systems could enhance autonomy among older adults with cognitive impairment by assisting with task management, providing memory supports, and fostering social engagement [21,25,29]. However, perceptions were not universally positive. Other studies reported concerns that robots might inadvertently undermine autonomy by imposing external control that could limit personal choice [12].

#### 3.1.2. Domain 2: Barriers to Adoption

Despite broad recognition of potential benefits, participants identified several barriers to the adoption of SARs and assistive robots for older adults with cognitive impairment. These barriers spanned ethical concerns, technical limitations, design issues, emotional disconnects, and cost.

##### Theme 1: Ethical Concerns and Emotional Disconnect

Ethical and privacy-related concerns were a prominent barrier across studies. Participants expressed discomfort with continuous monitoring, including situations where robots were perceived to be “watching” even when inactive [3]. Fears of surveillance, data misuse, and health information collection without proper consent were also reported among individuals with cognitive impairment and their caregivers [12,22,24,25]. Some participants questioned whether robots undermined user autonomy, especially when control of the device and its data rested with external parties [12,26].

Robots’ potential to reduce human contact and increase social isolation was also raised, especially when used in place of in-person caregiving [3,21,22,24,26]. Companion robots were sometimes mistaken for real entities, raising concerns about deception, especially when users were unaware or forgot that a human was remotely controlling the system [3,22,28]. These emotional disconnections were compounded by robots’ lack of facial expressions, eye contact, and genuine emotion, which some users saw as barriers to forming meaningful bonds [12,32]. Cultural insensitivity, stigma, and even potential for abuse, especially when robots were managed remotely by unfamiliar personnel, were also mentioned as ethical risks [3].

##### Theme 2: Technical Fears and Usability Challenges

Participants frequently expressed anxiety about robot installation, daily operation, and troubleshooting. Concerns about complexity, reliability, and unfamiliarity with internet-enabled devices were especially pronounced among individuals with advanced cognitive impairment [21,24–26,32]. Studies noted that a gradual, well-timed introduction was critical to user acceptance: robots introduced too early were seen as unnecessary, while those introduced too late were often rejected due to diminished cognitive capacity [3,21,32]. Technical fears also included concerns about malfunctions, poor reliability, and safety hazards, such as doors opening unexpectedly or the robot failing during emergencies [12,25].

##### Theme 3: Design and Functionality Limitations

Design-related barriers were widespread. Participants expressed preferences for smaller, more discreet robots suited to home environments [24,32]. Robots perceived as mechanical or toy-like, especially pet robots, were sometimes dismissed as frivolous or childish, particularly by individuals who had real pets [23,26]. Studies also emphasized the need for robots to sound and behave more naturally. Recommended improvements included more human-like voices, informal and culturally familiar language and accents, and adjustable volume for users with hearing impairment [31–33].

Functional limitations were also noted. Users criticized robots that moved too slowly, produced loud or jarring sounds, or failed to navigate environments effectively, contributing to tripping hazards or frustration [21,22,24,26,31]. Participants also expressed a need for better video prompt quality, larger screen displays, and enhanced ability for robots to initiate meaningful conversation [31,32]. Some caregivers reported a “dual burden of care,” where managing the robot added new responsibilities, although this was not universally observed [21,23,24,29].

##### Theme 4: Caregivers and Care Receiver Misalignment

Social dynamics also influenced robot acceptance. In dyadic studies, older adults with dementia were often influenced by caregiver preferences. Disagreements over the robot’s usefulness or appropriateness reduced acceptance, especially when care receivers deferred to their care partners [25]. Differences in expectations between caregivers, providers, and older adults with cognitive impairment, particularly around virtual communication features, also affected perceptions. For instance, providers valued remote visits for efficiency, while caregivers stressed the importance of in-person interaction to maintain social connection [24].

##### Theme 5: Cost Concerns

Cost was a frequently cited barrier. While some participants acknowledged the long-term economic potential of robotic assistance, high upfront costs of acquisition and maintenance were viewed as prohibitive. Moreover, many emphasized that robots could not fully replace human caregivers, complicating perceived return on investment [21,25,26,32]. As a result, cost concerns were often tied to value judgments about what care should involve—and what aspects of caregiving robots could, or could not, ethically and emotionally replace.

#### 3.1.3. Domain 3: Suggestions for Improving Robots for Independent Living

Participants across several studies provided recommendations for improving the usability, acceptability, and effectiveness of SARs and assistive robots for older adults with cognitive impairment. Suggestions spanned three primary areas: personalization and adaptation, enhancements to physical and interactive features, and the integration of advanced functionalities that support cognitive, emotional, and physical well-being.

##### Theme 1: Personalization and Adaptation

Personalization was a consistent priority. Several studies emphasized the need for individualized training based on users’ comfort with technology. Tailored onboarding processes were seen as critical to easing adoption and increasing confidence [21,24,25]. Customizing the robot’s functions and responses to the specific household context and user preferences was also recommended to reduce frustration and enhance users’ sense of control [12]. For example, adapting navigation capabilities to the home layout was suggested to improve mobility and efficiency within the environment [21].

##### Theme 2: Enhancing Robot Features

Participants also proposed concrete design improvements aimed at increasing emotional connection and trust. Realistic and familiar auditory features, such as more human-like voices, were recommended to reduce resistance and improve the robot’s relatability [21,22]. Participants also called for more reliable movement, faster response times, and alert systems that notify users before the robot moves, all of which could reduce user discomfort and foster greater trust [31].

##### Theme 3: Advanced Functionalities

Advanced functionalities were widely viewed as essential for promoting independence. Voice-command systems and emergency response buttons were endorsed across stakeholder groups, as they increase accessibility and responsiveness during critical situations [24,33]. Participants also recommended features that support memory and daily routines, such as reminders to take medication, recognize family members, or find misplaced items, as a way to preserve autonomy and social connection during cognitive decline [24,32,33]. The ability to break down daily tasks into small, manageable steps was another recurring suggestion, with users noting that such support could facilitate independent task completion and reduce frustration [31–33].

Finally, participants suggested incorporating cognitively and physically engaging features, such as interactive games, exercise prompts, and reminders for essential daily activities (e.g., paying bills), to help maintain both mental stimulation and functional independence [32,33]. These enhancements were viewed as particularly important for supporting long-term engagement for cognitive health maintenance.

### 3.2. Quantitative Findings

Quantitative synthesis incorporates evidence from standalone quantitative studies as well as the quantitative strands of mixed-methods research. Findings were organized by primary measures and, where applicable, further subdivided into specific dimensions to facilitate comparison across studies with diverse methodological approaches.

#### 3.2.1. Measure 1: Usability and Usefulness

Harris-Gersten et al. evaluated usability based on user engagement frequency and caregiver involvement in prompting use. Although 80% of individuals with dementia used the companion pet robot, 35% of caregivers actively reminded them to interact with it, which indicates moderate spontaneous engagement, with some reliance on caregiver prompting [23]. This study also found that 80% of participants perceived the robot as effective in reducing agitation among individuals with dementia, suggesting perceived therapeutic benefits. Dosso et al. reported that 62% of people with dementia and 77% of caregivers found the tested robots useful, although only 29% of individuals with dementia indicated they would use the robot if they owned one [22]. This suggests that perceived usefulness may not always translate into intention to adopt among individuals with dementia. Among the three pet-like robots assessed (i.e., JustoCat, AIBO, and MiRo), JustoCat received the highest perceived usefulness rating at 82%, likely due to its realistic design and behavior. Furthermore, 25% of participants believed the robot would be broadly beneficial for people with ADRD. In a study by Chen et al., high usability was demonstrated through sustained engagement with a humanoid robot, nearly 273 s of conversation on average and a mean attention rate of 62.5% across three participants [29]. Even though the study sample size was fairly small, the findings suggest that the robot successfully held the user’s attention in a controlled setting. Similarly, Yuan et al. reported that more than half of caregivers believed the robot could be highly useful in dementia care, indicating caregivers’ willingness to integrate assistive technology into care routines for people living with dementia [32].

#### 3.2.2. Measure 2: Acceptance and Satisfaction

Two studies directly examined satisfaction with robots. Harris-Gersten et al. found that over 80% of participants were satisfied with pet robots, indicating high acceptance [23]. Similarly, Dosso et al. reported that 50% of older adults with dementia believed the robot could make life more interesting [22]. However, skepticism about the robot’s emotional capacity was observed: 29% of those with dementia and 14% of caregivers doubted the robot’s ability to express emotions or understand others. Caregiver enjoyment was notably higher, with 73% reporting positive interactions, compared to 43% of individuals with dementia. In a comparative study by Raghunath et al., older and younger adults rated robots on several dimensions of user experience. On a 7-point scale, robots received a mean score of 5.31 (SD not reported) for likeability and 5.48 for cognitive demand, indicating participants found the robot pleasant to engage with and mentally stimulating [31]. These high scores indicate overall positive attitudes toward the system across age groups. Yuan et al. also found generally positive perceptions among individuals with ADRD [32]. For example, participants rated the robot’s physical appearance more positively (M = 4.5) than its voice (M = 3.61) on a 7-point Likert scale.

Dosso et al. further explored mutual stakeholder perceptions, finding that only 20% of individuals with dementia valued their caregiver’s opinion on the robot, whereas 79% of caregivers prioritized the opinions of their care recipients [22]. This disparity suggests a mismatch in perceived influence, with caregivers placing greater weight on the user’s perspective, which can affect adoption dynamics. Caregivers were also more likely than individuals with dementia to believe that friends and family would support robot use [21,22]. Harris-Gersten et al. reported a perceived benefit score of 1.35, above the benchmark of 1.0 on their custom scale, suggesting overall positive appraisals [23]. Caregivers noted that the robot benefited both the individuals with dementia (*n* = 13) and themselves. These dual benefits may contribute to higher caregiver endorsement. Furthermore, 89% of caregivers stated they would recommend the robot to others involved in dementia care, indicating strong perceived value among care partners. In a comparative study of younger and older adults, Raghunath et al. found that both groups reported high comfort using technology (≥5.8 out of 7), with no statistically significant difference, although older adults showed slightly more hesitation [31]. This similarity between the two populations suggests that comfort with technology may not be a major barrier to adoption in aging populations.

#### 3.2.3. Measure 3: Human–Robot Interaction

Cruz-Sandoval and Favela examined communication quality by comparing standard interactions to those employing sustained conversational strategies (SUcS) [34]. The SUcS approach led to a noticeable increase in participant utterances and sustained engagement, suggesting that conversational structure significantly improved engagement and communication frequency between the robot and individuals with dementia. Participants also reported high enjoyment during these enhanced interactions, which indicates emotional as well as functional benefits.

#### 3.2.4. Measure 4: Preferences

In terms of robot appearance and features, more than half of participants with dementia (56%) in one study preferred mobile robots over static ones [22]. Yuan et al. reported that 66 out of 79 individuals with ADRD found the robot’s height (approximately four feet) appropriate, and 66 out of 80 did not find the robot’s appearance frightening; only two reported it as “very scary” [32]. These results of Yuan et al. suggest that physical dimensions and neutral design features may support visual comfort and reduce intimidation. Acceptance of the robot’s appearance (M = 4.56) was rated higher than acceptance of its voice (M = 3.61) on a 7-point scale. These ratings show that while the robot’s visual presence was generally well received, auditory characteristics may require further refinement to enhance user comfort and appeal.

Guidance features also influenced preferences. Raghunath et al. found that “Next Step Video” and “Guide to Object” prompts were positively rated by participants with cognitive impairment, whereas full video guides were rated less helpful [31], which indicates that users prefer short, task-specific prompts rather than lengthy instructions, likely due to limitations in attention. Yuan et al. identified medication reminders, emergency call capabilities, and assistance in contacting medical services as the most favored features [32]. Participants preferred voice control over tablet-based interaction. However, in Turner and Berridge’s study, virtual companion robots were the least preferred option; only 5 out of 29 participants selected them over other technologies such as location tracking or in-home sensors [25]. This low preference (17.2%) indicates limited enthusiasm for socially assistive features when more practical or safety-focused ones are available. In contrast, 24 out of 29 participants expressed a strong preference for retaining control over technology, especially with regard to privacy and decision-making, indicating that autonomy is a critical factor in technology acceptance.

#### 3.2.5. Measure 5: Barriers

Low perceived need was a common barrier to adoption, especially among individuals with dementia who did not see a need for robotic assistance [22]. This perception may reflect a mismatch between user priorities and the functional offerings of available robots. Error tolerance and acceptance were also important factors. Raghunath et al. found that older adults with cognitive impairment demonstrated low tolerance for functional errors in robotic systems, which could negatively impact overall acceptance [31]. This sensitivity to malfunction could reduce user trust and discourage continued engagement. Robot appearance continued to influence perceptions. Unrealistic or overly artificial designs were associated with reduced acceptance, underscoring the need for relatable and familiar aesthetics [22].

## 4. Discussion

This review integrates qualitative and quantitative findings to offer a comprehensive understanding of how older adults with cognitive impairment, their caregivers, and healthcare providers perceive robots designed to support independent living. In addition, mixed-methods studies contributed insights to both strands of the synthesis, enriching the analysis by combining experiential perspectives with measurable outcomes. While qualitative and quantitative studies provide distinct perspectives, their combination yields a more holistic view of the potential benefits and limitations of robotic technologies. Overall, robots were generally viewed as beneficial; however, the data reveal that robot acceptance is complex, shaped not only by functionality, design, and the stage of an individual’s cognitive decline but also by deeper issues such as emotional bonding, ethical tradeoffs, and autonomy.

### 4.1. What Drives Robots’ Acceptance in Dementia Care?

Robot acceptance among older adults with cognitive impairment is not simply a matter of technical performance or novelty. Rather, it is more about how well these tools align with users’ emotional needs, cognitive ability, and perceived relevance to their everyday lives. Factors, such as robots’ features, appearance, and the introduction timing relative to the stage of dementia, were identified as critical for acceptance. Across studies, acceptance was significantly influenced by robot features; most participants expressed their desire for smaller robots with realistic and familiar appearances, as these features enhanced emotional connection and reduced discomfort [22,28,32,33]. In contrast, mechanical or toy-like designs were less favorably received. These findings are consistent with prior research on older adults, which shows that realism and familiarity, combined with adequate training, play a critical role in long-term acceptance [16,36,37].

However, appearance alone is not sufficient. Timing also emerged as a key factor. Introducing robots earlier in the disease trajectory was viewed as more effective, as later-stage cognitive decline reduced both the perceived need and ability to engage [21]. This suggests a window of opportunity where individuals still retain the capacity to learn, personalize, and incorporate robotic tools into their routines. A gradual, personalized introduction was seen as crucial, consistent with prior research emphasizing extended exposure and stepwise engagement to enhance acceptance [38,39].

Acceptance also varies based on perceived usefulness and emotional resonance. For example, JustoCat and SARs were rated highly by both individuals with cognitive impairment and caregivers [22,31,32] and were associated with neuropsychiatric improvements, such as reduced agitation and anxiety [21,23,28]. These effects align with findings from a systematic review by Hsieh et al., which found that SARs can reduce depression and anxiety, promote emotional well-being, and enhance social interaction [11].

Yet, the interpretation of acceptance must account for conflicting perspectives. Caregivers generally viewed robots more favorably, valuing practical features such as medication reminders and emergency call functions, while individuals with cognitive impairment were more ambivalent [22,24]. This disconnect can create tension in implementation; even well-designed robots may face rejection if users’ readiness and needs are overlooked. Notably, caregiver attitudes influenced acceptance. When caregivers endorsed the robot, individuals with cognitive impairment were more likely to accept it [25]. However, caregivers also expressed concern about the additional responsibilities introduced by robot management, echoing findings of a potential “dual burden” [21,23,24]. This points to a critical design implication; assistive robots should reduce, and not add to, caregiver workload. Centralized control systems may help reduce this strain and facilitate more seamless integration into care routines.

Technical barriers such as low error tolerance and digital unfamiliarity further complicate trust and acceptance [31]. In addition, reliability and usability are non-negotiable, particularly when users already face cognitive fatigue [12]. These findings suggest an important practical implication that onboarding protocols must be simple, adaptive, and culturally relevant. Tailored training and programs that offer extended exposure and person-centered training may help bridge this gap and support more confident use [21,29].

Finally, a pervasive barrier is the psychological distance between the user and the technology. Many individuals with cognitive impairment often expressed a low perceived need for robotic support, viewing themselves as not yet appropriate users [22,24,25,27]. This distance reflects both stigma and lack of self-identification with assistive tools. Similar patterns were observed by Wu et al., where individuals with MCI and intact cognition projected robot use onto “others” rather than themselves [40]. Therefore, interventions should incorporate messaging and framing strategies that normalize use and emphasize empowerment rather than dependency.

### 4.2. Emotional Bonding vs. Functional Utility

While assistive robots are often designed with functional tasks in mind, such as medication reminders and safety monitoring, the emotional connection aspect was also recognized as critical. A consistent theme in qualitative findings was the emotional bond formed with SARs, particularly companion robots and those that mimic pets. These bonds reportedly reduce loneliness, anxiety, and agitation, and value the companionship provided [21,22,25,26,28,30,33]. Quantitative findings supported these findings and reported emotional comfort from robot interaction [22,23]. Robots capable of sustaining longer, meaningful conversations were especially effective in fostering emotional engagement. Notably, robot conversational strategies significantly improved interaction and enjoyment among individuals with dementia [34]. These findings align with other studies showing that more “talkative” robots are perceived as more supportive and emotionally engaging [33,41–43]. However, emotional satisfaction does not guarantee adoption. While caregivers often value emotional benefits for their loved ones, some individuals with cognitive impairment were hesitant to use the robot consistently or see it as “for them”. This tension between emotional connection and fictional utility may be because they find it difficult to understand the robot’s capabilities, which presents an important consideration for designers to keep the balance between warmth and usefulness (practicality).

### 4.3. Tensions Between Safety, Surveillance, and Autonomy

The potential of robots to promote independence in dementia care is promising, yet this promise is challenged by persistent tensions around autonomy, surveillance, and user control. Robots offer valuable tools to promote independence, such as reminders, emergency functions, alerts, and cognitive prompts, by which users feel an enhancement in independence and confidence [21,25,29]. Quantitative studies, similarly, demonstrated moderate satisfaction with these features [24,31]. This aligns with prior literature emphasizing the potential of SARs and assistive robots to preserve autonomy in the face of cognitive decline [44–46]. However, perceptions of these technologies are not uniformly positive. Concerns emerged around over-reliance and loss of agency. Some users worried that robots might exert undue control or reduce opportunities for active engagement [12,26]. These findings underscore the importance of designing robots that support, rather than replace, human agency and decision-making and highlight a design dilemma of how to support users without undermining their autonomy. These tensions mirror broader debates in gerontechnology and demonstrate the need for transparency, user customization, and safeguards.

### 4.4. Ethical Concerns and Design Implications

Barriers identified across both qualitative and quantitative data included ethical concerns, technological complexity, unrealistic design, and low perceived need. Ethical concerns such as data privacy, informed consent, and the role of robots in reducing human contact were some of the most repeated concerns, all of which could influence adoption. Participants expressed discomfort with constant monitoring, potential data misuse, and reduced human interaction [3,12,22,24,25]. Informed consent emerged as a particular challenge, especially for individuals with progressive cognitive impairment. Establishing robust guidelines around data privacy, informed consent, and boundaries for robot autonomy will be essential for ethical implementation [40]. Addressing these issues requires ethical frameworks that respect dignity, define boundaries of autonomy, and ensure clarity in what robots can and should do.

The synthesis of qualitative and quantitative data reveals that while robotic technologies show promise in promoting independent living among older adults with cognitive impairment, their successful adoption depends on multiple factors. These include the robot’s perceived usefulness, ability to foster emotional connection, and capacity to support autonomy without displacing human control. Differences in perspectives between individuals with cognitive impairment and caregivers further underscore the need for personalized, context-aware robot design. Enhancements in usability, emotional interaction, and stakeholder involvement will be critical to improving satisfaction and long-term adoption.

Beyond these thematic findings, this review also contributes to the literature by integrating qualitative and quantitative evidence across multiple stakeholder groups (older adults with cognitive impairment, informal and formal caregivers, and healthcare providers). Earlier reviews often focus solely on older adults or single-method studies; in contrast, our synthesis provides a multidimensional perspective on perceptions of robots designed to support independent living, multiple valuable contributions to the field of assistive robots for living independently, and an underexplored context in dementia care. By also highlighting ethical and emotional dimensions such as autonomy, data privacy, and human–robot bonding, this review offers timely insights to guide the design and adoption of future robotic systems.

### 4.5. Limitations

This review has several limitations. First, we limited the search to peer-reviewed studies published in English, which may have excluded relevant evidence from non-English publications or the gray literature. Second, because the review focused on studies published between 2019 and 2024, earlier studies were excluded, and findings should be interpreted considering this time frame. Third, despite our use of a structured search strategy and predefined inclusion criteria, this review may be subject to interpretation bias. Finally, while we employed a convergent synthesis approach, our integration of qualitative and quantitative findings is necessarily interpretive and may not capture all individual studies.

### 4.6. Gaps in the Literature and Future Directions

Despite a growing body of evidence, critical gaps remain. Many included studies had methodological limitations, such as sample size, use of hypothetical or prototype-based exposure, and limited reporting of analytical procedures, which reduce the strength of inferences that can be drawn. These early-phase investigations contribute to the field’s development but may limit generalizability and ecological validity. We interpreted their data with appropriate caution and underscore the need for more rigorous, real-world evaluations in future research. To support wider adoption, future research should focus on developing robots that can adapt to the cognitive and emotional needs of users over time, including integrating health monitoring, personalized cognitive support, and ethically grounded data management. There are also limited data on tracking the use of robots beyond short interventions, making long-term effectiveness unclear. Thus, longitudinal studies are needed to examine how acceptance and use evolve across different stages of dementia. Stakeholders’ perspectives, especially from healthcare providers and under-represented groups, remain underexplored. Future research should include more diverse populations and examine the effect of provider perspectives and cultural contexts on robot adoption and care integration. Furthermore, accessible training and onboarding programs for both users and caregivers will be vital in ensuring equitable access and sustainable integration into care environments; intervention studies are needed to evaluate the impact of different training approaches on adoption and acceptance. Finally, establishing clear, inclusive guidelines for informed consent is critical, particularly as cognitive capacity declines. Future work should develop and test consent procedures tailored to progressive cognitive impairment.

## 5. Conclusions

This review synthesized qualitative and quantitative evidence to provide a comprehensive understanding of stakeholder experiences with assistive and socially assistive robots in the context of cognitive impairment and dementia care. More specifically, our study examined how people with cognitive impairment, their caregivers, and healthcare providers perceived and experienced interactions with robots. Our findings demonstrate that while these technologies show considerable promise in supporting independent living, emotional well-being, and caregiver assistance, their acceptance and effectiveness are influenced by multiple interrelated factors. As the global population ages and the demand for dementia care intensifies, there is a growing role for robotic technologies to complement human caregiving. However, successful integration will require more longitudinal studies and real-world implementation trials to assess sustained use and outcomes across diverse care settings. Incorporating caregiver perspectives will also be essential in the development of robotic solutions, as caregivers often act as both users and decision-makers in dementia care. The integration of robots into caregiving roles must be guided by evidence-based research, empathy, and stakeholder input from diverse people living with dementia and their care partners.

The findings indicate that perceived usefulness, emotional connection, and the ability to support autonomy emerged as critical determinants of acceptance. Robots with familiar and realistic designs, responsive conversational abilities, and functions tailored to user needs were generally viewed more favorably. Early introduction in the course of cognitive decline, along with gradual and personalized onboarding, was associated with higher engagement. Notably, differences in perception between individuals with cognitive impairment and caregivers highlight the importance of designing robots that can accommodate diverse expectations and experiences. Caregivers often emphasized practical utility, whereas individuals with cognitive impairment exhibited more ambivalence, particularly when emotional connection or understanding was limited. Barriers to adoption were evident across both data types and included concerns about privacy, ethical implications, technological complexity, and low perceived need. Users expressed discomfort with constant monitoring, limited trust in robotic reliability, and fear of reduced human interaction. Additionally, some participants raised concerns about over-reliance and potential loss of agency, suggesting the need for systems that support rather than supplant user decision-making and control.

## Supplementary Material

Tables S1-S4

**Supplementary Materials:** The following supporting information can be downloaded at: https://www.mdpi.com/article/10.3390/jal5030034/s1. Table S1: Keywords and Definitions; Textbox S1: Created PubMed Search String; Table S2: Eligibility Criteria for the Studies; Table S3: Search Queries for All Databases Searched; Table S4: Summary Overview of Themes.

## Figures and Tables

**Table 1.** Characteristics of reviewed studies.

Authors, Year	Country	Method	Framework	Robot Type and Function	Purpose	Participants	Data Collection and Analysis	Key Findings/Themes
Arthanat and Begum (2020) [21]	U.S.	Qualitative Design	UTAUT, technology acceptance	Socially assistive robot (SAR) Functions: reminding, alerting the care recipient	Beta-test a novel SAR with ADRD caregivers and gather perspectives on home integration.	Older adults *n* = 8; mean age: 78.5 Diagnoses: Alzheimer's (*n* = 7), Lewy body dementia (*n* = 1)	Observation, phone interviews, focus groups Analysis: grounded theory	Performance expectancy Effort expectancy Technology anxiety Perceived trust Facilitating conditions Social influence Cost expectancy
Dosso et al. (2022) [22]	Canada	Mixed-Methods Design (Survey + Open-Ended Questions)	TAM, Almere model, ACT	Three pet-like social robots: AIBO (dog-like) Joy for All Cat (JustoCat) MiRo-E (neutral animal) Functions: responding to petting, tricks, movement/sound	Understand emotional alignment and attitudes of (1) healthy older adults, (2) older adults with dementia, (3) care partners, in response to pet-like robots.	*n* = 206 participants Healthy older adults (*n* = 171) Care partners (*n* = 28) Persons with dementia (*n* = 7; mean age: 70)	Survey with open-ended questions Analysis: content analysis (qual) and descriptive analysis (quant)	Usefulness and intention to use Enjoyment and attitude Social interaction Movement and appeal Ethical concerns Emotional impact ACT-based modeling
Harris-Gersten et al. (2023) [23]	U.S.	Mixed-Methods Design	NIH stage model for behavioral interventions	Analysis: social robot pets (dogs and cats) Function: promote social engagement	Assess the usability and acceptability of robot pets among veterans with dementia and caregivers.	Veterans with dementia (*n* = 20; mean age: 81) Informal caregivers (demographics not specified)	Pre/post questionnaires; Structured phone interviews; video-recorded observations Analysis: content and descriptive analysis; paired *t*-test	Relaxation and comfort benefits Value of past pet experiences Caregiver support and positive reactions
Portacolone et al. (2020) [3]	U.S.	Case Study (Qualitative)	Biomedical ethics (respect, beneficence, non-maleficence, justice)	Telepresence pet-avatar robot Functions: emotional support (greetings, music); instrumental support (reminders, help with phone)	Describe ethical issues in a specific case using a pet-avatar robot and suggest improvements for ethical application.	2 providers 1 developer	Field notes from seminar; interviews; review of articles Analysis: content analysis	Deception Monitoring and tracking Informed consent Social isolation
Sawik et al. (2023) [12]	Poland	Qualitative	Person-centered care	Mobile service robot	Explore robotic support for older adults with MCI.	Older adults with MCI (*n* = 8; mean age: 76.5) Informal caregivers (*n* = 5; mean age: 42.1) Formal caregivers (*n* = 5; mean age: 45.6)	Focus groups Analysis: grounded theory	User characteristics Robot characteristics Robot functions Barriers
Shin et al. (2022) [24]	U.S.	Qualitative Design	TAM (perceived usefulness, ease of use)	Mobile telepresence robot Functions: two-way video communication; remotely controlled movement	Assess applications to support the independence of individuals with MCI or ADRD and gather stakeholder opinions.	People with dementia (*n* = 6; mean age: 73) Caregivers (*n* = 9; mean age: 60) Clinicians (*n* = 6)	Semi-structured interviews Analysis: thematic analysis	Technology use Robot design and functionality Future applications Opinions, concerns, perceived value
Turner and Berridge (2023) [25]	U.S.	Mixed-Methods (Convergent Design)	H-TAM	Virtual companion robot Function: talking and listening	Examine the technology choices of people with mild AD and fit with the H-TAM framework.	Older adults with mild AD (*n* = 21; mean age: 70)	Dyadic interviews via Zoom/in-person Analysis: thematic and bivariate analysis	Technology preferences Ease of use Usefulness Autonomy and privacy concerns
Van Assche et al. (2022) [26]	Belgium	Qualitative Design	COREQ	Socially assistive robots (SARs) Functions: talking, moving, playing music	Explore attitudes of older adults and caregivers toward SARs in assisted living.	Older adults with MCI (*n* = 30; mean age: 82) Caregivers (*n* = 10; mean age: 52)	In-depth interviews Analysis: grounded theory (inductive)	Perspectives on robot use Implementation views Differences by stakeholder group
Van Assche et al. (2024) [27]	Belgium	Qualitative Design	COREQ	SARs with autonomous navigation Functions: voice recognition, entertainment, cognitive games	Explore needs and preferences regarding SARs in assisted living.	Older adults with MCI (*n* = 30; mean age: 82) Caregivers (*n* = 10; mean age: 52)	Semi-structured interviews Analysis: thematic analysis	Companion Cognitive assistant Coach and leisure buddy Health, household, and job support
Van Orden et al. (2022) [28]	U.S.	Qualitative Design	None specified	Joy for all pets (puppies, cats) Functions: respond to touch, voice, blinking, and meowing	Inform implementation of robotic pet programs in real-world dementia care.	Veterans with dementia (*n* = 9; mean age= 81) Staff (*n* = 8)	Staff observations Analysis: descriptive analysis	Neuropsychiatric and social benefits Challenges of implementation
Chen et al. (2020) [29]	Taiwan	Case Study (Mixed Methods)	None specified	Teleoperated humanoid robot Function: social interaction	Explore participant experience, NPS impacts, uncanny valley response, and influencing factors.	Older adults with major NCD (*n* = 3) Ages 78–86	Observation and scoring Analysis: descriptive + qualitative notes	Eye contact Speech Emotional response Neuropsychiatric symptoms
Coşar et al. (2020) [30]	UK, Greece, Poland	Qualitative	None specified	Assistive robot (ENRICHME) Functions: object location, games, tracking	Evaluate user interaction and experience with ENRICHME.	Older adults from UK (*n* = 3), Greece (*n* = 4), Poland (*n* = 4) Ages 66–90+	Observation, interviews Analysis: mixed; qualitative method not specified	Emotional connection Acceptance Design and functionality feedback
Raghunath et al. (2020) [31]	U.S.	Mixed Methods	None specified	Robot activity support (RAS) Functions: assist with ADLs, detect people/objects	Compare perceptions of RAS between younger and older adults.	Younger adults (*n* = 26) Older adults with dementia (*n* = 26)	Surveys with open-ended questions Analysis: descriptive + thematic	Comfort Design suggestions Voice/sound preferences
Yuan et al. (2022) [32]	U.S.	Mixed Methods	Almere model	Pepper (SoftBank humanoid robot) Functions: reminders, entertainment, exercise, monitoring	Assess attitudes toward Pepper for ADRD care and identify user needs.	Older adults with dementia (*n* = 80) Caregivers (*n* = 245) General public (*n* = 306)	Online survey with open-ended questions Analysis: descriptive, Mann-Whitney, word cloud	Robot appearance and voice Functionality and impact Intention to use Ethical concerns
Law et al. (2019) [33]	New Zealand	Qualitative Design	None specified	Silbot (semi-autonomous) Functions: safety, health monitoring, and cognitive activities	Evaluate the usefulness of daily care functions shown via the robot.	Older adults with dementia (*n* = 9) Providers (*n* = 19 total across two studies)	Semi-structured interviews Analysis: descriptive qualitative	Robot interaction Appearance and functionality Suggestions for improvement
Cruz-Sandoval and Favela (2019) [34]	Mexico	Quantitative Methods	None specified	Ava (SAR using Wizard of Oz) Functions: conversation, emotion display, music	Assess effectiveness of conversation strategies to enhance PwD communication.	Older adults with dementia (*n* = 12; mean Age: 80)	Video recording Analysis: coding, *t*-test, visual analysis	Engagement Enjoyment Conversation duration and quality

Note: TAM = Technology Acceptance Model; SAR = Socially Assistive Robot; UTAUT = Unified Theory of Acceptance and Use of Technology; ACT = Affect Control Theory; H-TAM = Healthcare Technology Acceptance Model; COREQ = Consolidated Criteria for Reporting Qualitative Research; NPS = Neuropsychiatric Symptoms; NCD = Neurocognitive Disorders; PwD = People with Dementia.

## Data Availability

The original data presented in the study consist of five databases: PubMed, Web of Science (all collections), CINAHL (Ebsco), MEDLINE (Ebsco), and Scopus, which are openly available at https://clemson.libguides.com/az/databases (accessed on 11 July 2025).

## Abbreviations

The following abbreviations are used in this manuscript:

ADRD: Alzheimer’s disease and related dementias
PICo: Population, Phenomenon of Interest, Context
MCI: Mild cognitive impairment
AI: Artificial intelligence
