# Supplementary material for: Independent Living for Older Adults with Cognitive Impairment: A Narrative Review of Stakeholder Perceptions and Experiences with Assistive and Socially Assistive Robots
Source: J Ageing Longev. Author manuscript; Available in PMC 2026 Jun 12. (PMC13253492; doi:10.3390/jal5030034)
Supplement: Tables S1-S4 [file NIHMS2178994-supplement-Tables_S1-S4.docx]

**Table S1. Keywords and Definitions**

| **Keyword** | **Definition** |
| --- | --- |
| Aged | A person 65 years of age or older. For a person older than 79 years, AGED, 80 AND OVER is available. Year introduced: 1966  “Aged” [Mesh] or “Frail Elderly”* [Mesh] |
| Dementia | A degenerative disease of the BRAIN characterized by the insidious onset of DEMENTIA. Impairment of MEMORY, judgment, attention span, and problem-solving skills are followed by severe APRAXIAS and a global loss of cognitive abilities. The condition primarily occurs after age 60 |
| Robotics | The application of electronic, computerized control systems to mechanical devices designed to perform human functions. Formerly restricted to industry, but nowadays applied to artificial organs controlled by bionic (bioelectronic) devices, like automated insulin pumps and other prostheses.  Year introduced: 1987  “Robotics”[Mesh] |
| Independent Living | A housing and community arrangement that maximizes independence and self-determination.  Year introduced: 2010  “Independent Living”[Mesh] |

* The terms in quotation marks are direct quotes from MeSH.

**Textbox 1. Created PubMed Search String**

| **(("frail elder*"[tw] OR "Aged"[tw] OR "elderly"[tw] OR "elderlies"[tw] OR "elderly s"[tw] OR "elderlys"[tw] OR "Aged"[MeSH Terms]) AND ("soft robotic*"[tw] OR "Robotics"[MeSH Terms] OR "Robotics"[tw] OR "telerobot*"[tw] OR "telerobots"[tw] OR "socially assistive robot*"[tw] OR "social robot*"[tw] OR "humanoid robot*"[tw] OR "remote operation robotic*"[tw] OR "companion robot*"[tw])) AND ("independent living"[tw] OR "community dwelling*"[tw] OR "Aging in Place"[tw] OR "independent living"[MeSH Terms])) AND (Alzheimer OR "Alzheimer's" OR dementia OR "cognitive impairment" OR cognition OR cognitive OR "Alzheimer Disease"[MeSH Terms])** |
| --- |

Note: All terms noted in this box are direct quotes from MeSH.

**Table S2. Eligibility Criteria for the Studies**

| **Criteria** | **Inclusion** | **Exclusion** |
| --- | --- | --- |
| Participants | Older adults aged 50 and above with cognitive impairment, formal and informal caregivers, Healthcare providers | Older adults with healthy cognitive ability |
| Phenomena of interest | The experiences and perceptions about robots that promotes independent living | The focus is evaluating the robotic system as opposed to users’ experiences |
| Context | Settings include older adults’ home or home-like labs or labs with a focus on independent living design | Focus on healthcare robots that were designed to be used in institutions, nursing homes |
| Types of Studies | Qualitative studies, Mixed-methods studies, Survey research, peer-reviewed studies, Published after January 2020 in English Language | Reviews |

Table S3. Search Queries for All Databases Searched

| **Search String** | **Keywords** | **Results #** |
| --- | --- | --- |
| PubMed | **(("frail elder*"[tw] OR "Aged"[tw] OR "elderly"[tw] OR "elderlies"[tw] OR "elderly s"[tw] OR "elderlys"[tw] OR "Aged"[MeSH Terms]) AND ("soft robotic*"[tw] OR "Robotics"[MeSH Terms] OR "Robotics"[tw] OR "telerobot*"[tw] OR "telerobots"[tw] OR "socially assistive robot*"[tw] OR "social robot*"[tw] OR "humanoid robot*"[tw] OR "remote operation robotic*"[tw] OR "companion robot*"[tw])) AND ("independent living"[tw] OR "community dwelling*"[tw] OR "Aging in Place"[tw] OR "independent living"[MeSH Terms])) AND (Alzheimer OR "Alzheimer's" OR dementia OR "cognitive impairment" OR cognition OR cognitive OR "Alzheimer Disease"[MeSH Terms])** | 34 |
| CINAHL (Ebsco) | (("frail elder*OR "Aged"" OR elderly OR elderlies OR "elderly s" OR elderlys OR (MH Aged+)) AND ("soft robotic*" OR (MH Robotics+) OR Robotics OR telerobot* OR telerobots OR "socially assistive robot*" OR "social robot*" OR "humanoid robot*" OR "remote operation robotic*" OR "companion robot*") AND ("independent living" OR "community dwelling*" OR "Aging in Place" OR (MH "independent living+")) AND (Alzheimer OR Alzheimer's OR dementia OR "cognitive impairment" OR cognition OR cognitive OR (MH "Alzheimer Disease+")) | 19 |
| MEDLINE (Ebsco)  the query is the same as CINAHL | (("frail elder*OR "Aged"" OR elderly OR elderlies OR "elderly s" OR elderlys OR (MH Aged+)) AND ("soft robotic*" OR (MH Robotics+) OR Robotics OR telerobot* OR telerobots OR "socially assistive robot*" OR "social robot*" OR "humanoid robot*" OR "remote operation robotic*" OR "companion robot*") AND ("independent living" OR "community dwelling*" OR "Aging in Place" OR (MH "independent living+")) AND (Alzheimer OR Alzheimer's OR dementia OR "cognitive impairment" OR cognition OR cognitive OR (MH "Alzheimer Disease+"))) | 40 |
| Web of Science | ((“frail elder*” OR “Aged" OR elderly OR elderlies OR "elderly s" OR elderlys OR Aged) AND ("soft robotic*" OR Robotics OR Robotics OR telerobot* OR telerobots OR "socially assistive robot*" OR "social robot*" OR "humanoid robot*" OR "remote operation robotic*" OR "companion robot*") AND ("independent living" OR "community dwelling*" OR "Aging in Place" OR "independent living") AND (Alzheimer OR Alzheimer's OR dementia OR "cognitive impairment" OR cognition OR cognitive OR "Alzheimer Disease")) | 109 |
| Scopus | (("frail elder*” OR “Aged" OR elderly OR elderlies OR "elderly s" OR elderlys OR Aged) AND ("soft robotic*" OR Robotics OR Robotics OR telerobot* OR telerobots OR "socially assistive robot*" OR "social robot*" OR "humanoid robot*" OR "remote operation robotic*" OR "companion robot*") AND ("independent living" OR "community dwelling*" OR "Aging in Place" OR "independent living") AND (Alzheimer OR Alzheimer's OR dementia OR "cognitive impairment" OR cognition OR cognitive OR "Alzheimer Disease")) | 63 |

Note: All terms noted in this box are direct quotes from MeSH.

Table S4. Summary Overview of Themes

| **Themes** | **Subtheme** | **Definition** | **Quote** |
| --- | --- | --- | --- |
| **User Perceptions and Experiences** | | | |
| Perceived Usefulness and Benefits |  | The subjective perception of users that using robots can improve their independent life and the positive outcomes or advantages that individuals believe they will gain from using the robot which can influence their willingness to adopt it. | *“Mostly I dislike their limited capacity for interaction or usefulness.” “Not as many features as I’d like.”* (Dosso et al., 2022) |
|  |  |  | *“[O]n an average I’ll be honest with you I avoid the doctor as much as possible. I avoid them like the plague because it’s something I don’t like—the smell of hospitals. They make me sad and they’re a depressing place. That’s another reason I don’t like going...[I]f I can avoid going to the doctor and I can just sit at home and say [to the doctor] see this, [the robot would help].”* [Person F with MCI or ADRD] (Shin et al., 2022) |
|  |  |  | *“[I]f there was a button that [people] could just push on the [robot] itself. [The robot] would have programmed in your address and how to get into the house for the emergency responders. And if it could automatically open a door, if the doors are locked.”* [Person B with MCI or ADRD] (Shin et al., 2022) |
|  |  |  | *“I think that a lot of the things that it’s used for can already be done through Facetime or Skype or what not. I think that there’s a little bit extra better quality to this.”* [Clinician C] (Shin et al., 2022) |
|  |  |  | *“Her early memory is kind of fading. But that’s where she’s at so I don’t see having a robot on hand would be a benefit to myself or her.”* (Arthanat and Begum, 2022) |
|  |  |  | *“No, I wouldn’t need it yet. If I become more dependent, then I think about it…I’m not against it [the idea of having robot] but it’s not necessary yet.”* (Van Assche et al., 2022) |
|  | Safety and Peace of Mind | The sense of security individuals feels when interacting with or being around the robot and the emotional reassurance and comfort they experienced knowing the robot could help or monitor. | *“Really to be able to see her all the time [is the greatest value]. To see what she’s doing because I’ve gone to the house and the stove is on. I’m like why is the stove on? I don’t know. I’m like okay we need to be able to see her all the time.”* [Family Caregiver D] (Shin et al., 2022) |
|  |  |  | *“[T]he security feature. So often we hear someone falls or something very minor escalates, like they leave a potholder on the stove and turn and walk away and the potholder burns and there’s a kitchen fire. And it’s not even necessarily the gravity of the act. I think that it’s the fear of what could happen ends up curtailing the person’s independence...[T]he daughter or son being able to say [using the robot], what’s that box doing in the middle of the floor? Move that to the right. [This] could prevent a fall.”* [Clinician B] (Shin et al., 2022) |
|  |  |  | *“I’m free to go out when I have an aide at home, but I’m not sure if I can leave him (care recipient) with the robot all alone.”* (Arthanat and Begum, 2022) |
|  |  |  | *[The robot would give] “a sense of security when one is lonely (. . . ) because I would like to wake up thinking that one is already there; one would feel less lonely with a robot.”* (Sawik et al., 2023) |
|  |  |  | *“It would give them a regularity that would take a huge amount of anxiety away at the beginning of each day.”* [Person with ADRD] (Law et al., 2019) |
|  |  |  | *“All you have to do is call out help and the robot will come. I think that is very clever. In fact, the whole robot scenarios are very clever.”* [Provider] (Law et al., 2019) |
|  | Household Assistance | The support provided by the robot in performing or aiding with daily household tasks, which can reduce the workload or physical effort required by the individuals. | *[Robots should do] “Anything that’s helpful for humans.” “Fetch and carry.”* (Dosso et al., 2022) |
|  |  |  | *“Prepare a meal, perhaps. Actually, anything you would otherwise do yourself, like doing the dishes.”* (Van Assche et al., 2024) |
|  |  |  | *“Make some coffee, make some sandwiches. […] Things you can no longer do yourself. If you are not well on your feet and you have to use your cane for everything… Also, you can’t get a cup of coffee with two crutches. That’s the idea, to have a little helper.”* (Van Assche et al., 2024) |
|  | Physical and Cognitive Assistance | The support offered by the robot to help with memory, organization, or other cognitive tasks, aimed at enhancing the individual’s ability to manage daily activities and reduce mental strain along with support for physical activities such as reminders to perform daily exercises. | *“Kuba brings a lot of variety. Everyone is interested, more willing to visit [...] the games are very interesting, I really like the puzzle one. The atmosphere at home is more revived by this robot.”* (Coşar et al., 2020) |
|  |  |  | *“It would be a good opportunity to keep our health standards up a bit, and physical exercise is an important part. So, if he [the robot] can get involved in this, we'll be more inclined to do exercises.”* (Van Assche et al., 2024) |
|  |  |  | *“Activities can be done in a playful way. In such a way that it might be less boring than doing the real exercises.”* [Caregiver] (Van Assche et al., 2022) |
|  |  |  | *“Keeping my memory up to date. So, reminding me of things I need to do. That’s my weakness, my short-term memory. For example, I forgot that you were coming today [for the interview]. That sort of things would be most useful, I think.”* (Van Assche et al., 2024) |
|  |  |  | *“Maybe questioning their daily routines. Just like when the nurse would come: “have you taken your pills already?”, or “have you eaten?”. Or if the afternoon is approaching, “it’s time to cook the potatoes “or “what are you going to cook?”* (Van Assche et al., 2024) |
|  | Emotional and Social Support | The comfort and companionship provided by the robot, which can help alleviate feelings of loneliness, provide emotional reassurance, and promote a sense of connection. | *“I think [the robot] would offer companionship because like I said I don’t have any family really. I don’t have any friends at all so I’m kind of alone all the time. It doesn’t really bother me. I get used to it after a while. But yeah, I think for somebody like me, [the robot] would offer companionship.”* [Person F with MCI or ADRD] (Shin et al., 2022) |
|  |  |  | *“It's also something that challenges them. [...]. It’s something to engage themselves with, it also provides some company.”* [Caregiver] (Van Assche et al., 2022) |
|  |  |  | *“He [the robot] can actually become a buddy, a friend that they can talk to a lot. And who also talks back. There is companionship, where they [the older adults] can also ask questions such as ‘have I already taken my medication?’ or ‘what day is it?’”.* [Caregiver] (Van Assche et al., 2024) |
|  |  |  | *“I think they will mostly feel like somebody is there for them and somebody is caring for them even though it’s not a human being. But they have someone to interact with which is quite good.”* [Provider] (Law et al., 2019) |
|  | Health-related Features and Emergency Assistance | The robot’s ability to detect and respond to emergencies, providing immediate support or alerting caregivers in situations like falls or medical crises as well as functionalities of the robot designed to monitor, support, or manage aspects of physical health | *“[T]he security feature. So often we hear someone falls or something very minor escalates, like they leave a potholder on the stove and turn and walk away and the potholder burns and there’s a kitchen fire. And it’s not even necessarily the gravity of the act. I think that it’s the fear of what could happen ends up.”* (Shin et al., 2022) |
|  |  |  | *“alerted me right away that I missed a step in the activity”* [Person with ADRD] (Raghunath et al., 2020) |
|  |  |  | *“A robot that is aligned to your health. It should take your blood pressure and everything. And if things are possibly wrong, that he reports it immediately.”* (Van Assche et al., 2024) |
|  |  |  | *“If you faint and the ambulance comes, it could tell the doctor what is wrong with you, it could have your medical history inside.”* (Sawik et al., 2023) |
|  | Pandemic and Workforce Shortage | The utilization of robots to address gaps in caregiving workforce, helping to meet care needs when there is a shortage of available human staff. | *“With COVID for starters . . . you can have a caregiver that you really like taking care of your person and all of the sudden they’re down with COVID and they’re out for two weeks and you’re scrambling to replace them. Whereas some of these technologies would be probably a little more reliable.”* [Care partner] (Van Assche et al., 2022) |
|  |  |  | *“I think that's a good thing, because the staff is too expensive […]. But actually, I 272 think humans are still better than robots. Because they can still think.”* (Van Assche et al., 2022) |
|  |  |  | *“I think it's good that, if there is no human guidance anymore, he [the robot] can take over some tasks. […] And he will probably forget less than a human. […]. I think it is a 276 valuable addition when there is a shortage of people to execute the job.”* (Van Assche et al., 2022) |
|  | Who Benefit the Most | Identification of the specific groups or individuals, such as older adults with limited mobility or cognitive impairments, who experience the greatest advantages from robot assistance in their daily lives. | *"To me, the underserved, under-cared for sector is the lower income sector. Those with little to no family. I believe they would benefit the most from this type of technology."* (Yuan et al., 2022) |
|  |  |  | *“This won’t be of much use to us older group, but it will for the next generation coming along; This sort of technology needs to be developed now, so the next generation of caregivers can readily take advantage of it.”* (Arthanat and Begum, 2022) |
| Acceptance and Satisfaction |  | The extent to which individuals are willing to adopt or use the robot and the degree of contentment or positive feelings expressed by the individuals regarding their experiences with the robot |  |
|  | Appearance and Realism | Realistic design of the robot with a more human-like or familiar appearance to enhance comfort and relatability, making interactions feel more natural and realistic. | *“Most similar to a living animal.” “Lifelike.”* (Dosso et al., 2022) |
|  |  |  | *“…the fur it has makes me want to pet it.” “The feel of the fur is comforting.”* (Dosso et al., 2022) |
|  |  |  | *"Developing a more human sounding voice is needed. The machine/computer voice would be extremely startling to a patient."* (Yuan et al., 2022) |
|  |  |  | *“I think it is good that it does not resemble a human being, that it actually looks like a machine, because if it had limbs, even immobile ones, it would be scary”* [B] *“I was thinking about some fur, the robot can be rendered more humanoid, dressed, decorated.”* (Sawik et al., 2023) |
|  |  |  | *“I really liked the arm movement. The before bed tap on the head was really nice. It really humanises it in a really interesting way”* [Provider] (Law et al., 2019) |
|  | Engagement and Interaction | To what extent the robot is encouraging and stimulating active interactions with users. | *“An element of interaction that would add color to your day.”* (Dosso et al., 2022) |
|  |  |  | *“Maybe it [the SAR] could play a game. Cards, trivia questions; “Staying connected with their old life. They get very bored, they get lonely. Something interactive. That’s vital.”*  (Arthanat and Begum, 2022) |
|  |  |  | *“I think loneliness is the big issue, so I had suggested if it could interact like an interactive game with the person, or you know, have a short conversation maybe play some music.”* (Arthanat and Begum, 2022) |
|  |  |  | *“The magic show was wonderful and Beauregard with his little tuxedo and top hat was a hit. Every time the resident spoke or the audience clapped, Beauregard (responding to the noise and movement) would turn his head and bark a happy yip.”* (Van Orden et al., 2022) |
|  | Caregiver’s/Care-receiver Acceptance | The extent to which caregivers or care-receiver view the robot as beneficial and are comfortable integrating it into the care environment, impacting overall adoption and satisfaction. | *“If I can control [the robot] from afar that's helpful. If it can do some things on its own like little reminders and things, that's helpful. It just serves as a bridge and the fact…like I said for my dad where he doesn't trust humans to come into the house maybe he would let a machine.”* [Family caregiver C] (Shin et al., 2022) |
|  |  |  | *I don’t have time for extra things that take time if it gets too complicated; Are we going to spend more time playing with it to try and make it run? Then we are caretaking it too.”* (Arthanat and Begum, 2022) |
|  |  |  | *I’m confident that it [the robot] will do what it is supposed to do, but I’m not sure if my husband will follow through.”* [Care partner] (Arthanat and Begum, 2022) |
|  |  |  | *“Everyone across generations in my family will be supportive, I think they would think it’s a good idea…they would be very favourable of having anything that makes things easier for me.”* (Arthanat and Begum, 2022) |
| Emotional Connection |  | The bond or attachment individuals feel toward the robot, often resulting in a sense of companionship or attachment, which can positively impact their emotional well-being. | *“They [the robot] offer company and a reason to direct love.”* (Dosso et al., 2022) |
|  |  |  | *“One Veteran in particular has become extremely connected to his puppy and feeds her ‘people food.’ . . . A few weeks ago, I was visiting him in his room and he said, ‘You’ve met my pup right? She’s napping right now otherwise I’d introduce you.’ He was in his wheelchair facing his puppy who was laying on his bed, little head on the pillow, all covered up with a blanket . . . His fixation with the ‘worms’ on his head (a psychological symptom for him) has decreased which is great too.”* [Nurse] (Van Orden et al., 2022) |
|  |  |  | *“He uses it every night. It makes him laugh and smile every time. He doesn’t remember its name, but he talks to it and loves on it before we fall asleep.”* [Caregiver] (Harris-Gersten et al., 2023) |
| Control and Autonomy |  | The extent to which individuals feel that using the robot allows them (or their care recipients) to maintain independence and make their own choices in daily activities, without feeling overly dependent or restricted by the robot's presence. | *“If someone sponsored such a robot to me, I would have a feeling that I was under control.”* (Sawik et al., 2023) |
| ***Barriers to Adoption*** | | | |
| Ethical Concerns and Emotional Disconnect |  | Considerations and potential dilemmas related to privacy, consent, dependency, and the moral implications of using robots in caregiving or personal spaces, which may affect individuals' comfort and acceptance.  Emotional disconnect, defined as difficulties individuals may face in forming a meaningful bond or sense of attachment with the robot, often due to its perceived lack of human qualities or emotional responsiveness, also influences user comfort, trust, and acceptance. | *“For some older adults they might replace social relationships and encourage further social isolation.” “They should not be… a substitute for human care and concern.”* (Dosso et al., 2022) |
|  |  |  | *“[F]or the most part, he would appreciate it although there are probably moments where it may feel intrusive...[need to make the robot] so it’s not intrusive into any person’s private moments...I think that’s the only thing I would think about if someone’s like, Hi, daughter, didn’t realize you were right here.”* [Family Caregiver F] (Shin et al., 2022) |
|  |  |  | *“I’m trying to picture a sort of dual control of the on/off [which would help with] the privacy concern. I mean the problem is that it helps him with the privacy concern but it doesn’t help me with the check on him. Cause he is liable to turn it off and forget that he turned it off and then if I can turn it on anytime I need to or want to—how does that give him the privacy that he is looking for. So there’s that tension there.”* [Family Caregiver B] (Shin et al., 2022) |
|  |  |  | *"...I am afraid too many families might place a relative with AD or dementia under the long-term care of a robot...."* (Yuan et al., 2022) |
|  |  |  | *“With [one Veteran] yesterday he was extremely upset because he thought his dog “died”. He told nursing “We’ve got to get a shovel, she hasn’t moved in an hour”. The batteries had died so nursing and myself took the puppy to the “hospital”. We put in new batteries and gave the dog a “bath” (washed fur up), took her to the “dentist” (cleaned out the food in the mouth) and returned her to XY who was overjoyed.”* [Social worker] (Van Orden et al., 2022) |
|  |  |  | *“Who makes this? Amazon? And where is it connected? Is it connected to the cell phone system and the internet? Okay, who’s listening? I could see hey there’s just a lot of issues here. And trust and so on.”* [Care partner] (Turner and Berridge, 2023) |
|  |  |  | *“Of course, it should not hinder them from going outside less or so. That they have such a good time with the robot that they feel less of a need for social contact. […]. Maybe people will visit less because they feel like, 'our task is taken over a bit'.* (Van Assche et al., 2022) |
|  |  |  | *“It’s slightly on purpose that the disclosure is slightly vague. We leave it a little bit to the caregiver how they want to disclose it…clearly, it’s a gray area.”* [Developer] (Portacolone et al., 2020) |
| Emotional Connection Challenges |  | Difficulties individuals may face in forming a meaningful bond or sense of attachment with the robot, often due to its perceived lack of human qualities or emotional responsiveness. | *“He doesn’t really use it, because he had a real dog that he loved so much. He knows this one isn’t real so he just kind of laughs at it, but doesn’t use it.”* (Harris-Gersten et al., 2023) |
|  |  |  | *“incapable of recognizing pain or needs”* (Van Assche et al., 2022) |
| Technical Fears and Usability Challenges |  | The apprehensions or anxieties individuals may feel regarding the robot, such as concerns about safety, malfunction, loss of control, or using technology. | *“Loud barks, noises could startle or scare some older adults”* (Dosso et al., 2022) |
|  |  |  | *“I don’t really use [the internet] that much. I’m not interested and it’s confusing.”* [Person A with MCI or ADRD] (Shin et al., 2022) |
|  |  |  | *“I think my mother would be good with [the robot]. I think my father, it might be challenging for him only because I don’t feel that he has the attention span. I think that maybe if this machine was in front of him and he saw what it could do, I think it might spark a little interest.”* [Family Caregiver E] (Shin et al., 2022) |
|  |  |  | *"I can’t imagine an Alzheimer’s patient having anywhere near the cognitive or emotional ability to LEARN to use any of the functions, if learning such things is necessary."* (Yuan et al., 2022) |
|  |  |  | *“I’m confident it will work the same way every time once it is set up right; the only anxiety is if it stops working; if it had a good technical crew, I will have less anxiety;” “a designated tech company has to help with set up and support.”* (Arthanat and Begum, 2022) |
|  |  |  | *“I'd rather not have him [the robot] with me, but if that's the only option... it’s like you then confess to your environment that you’re having problems.[…]. It [a robot] is obvious. When you meet people, you have to talk to them for a while before you realize there’s something going on. And a robot is standing right next to you.”* (Van Assche et al., 2022) |
|  |  |  | *[indicating fear of stigma]: “To me, it would be something personal. I wouldn't do that, walking around with it [the robot]”.* (Van Assche et al., 2022) |
|  |  |  | *“That's what scares me the most, it's still a robot. He can suddenly shut down, he can have bugs,... At this moment, I will never trust it 100%. It's still a machine, isn't it?”* (Van Assche et al., 2022) |
|  |  |  | *“This generation did not grow up with those objects, those modern techniques. […]. It can be stressful [for the older adults], I think. They're going to be afraid to touch something wrong, or press the wrong buttons.”* (Van Assche et al., 2022) |
|  |  |  | *“They forget quickly. If someone explains to them how the robot works, they will forget about it the next day. […]. They even forget how to use the microwave.”*(Van Assche et al., 2022) |
|  |  |  | *“Of course, people shouldn't get lazy... It offers something, but also takes something away at the same time.”* (Van Assche et al., 2022) |
|  |  |  | *“would it let a thief in?”[*6] *and an observation, “someone who has been let in could damage the robot.”* (Sawik et al., 2023) |
|  |  |  | *“Technical difficulties would make a person with dementia pretty confused so it would need to be able to run quite smoothly.”* [Provider] (Law et al., 2019) |
|  | Introduction to the Robot | The approach and timing of introducing the robot to an older adult with dementia, considering factors such as the stage of the disease and specific needs. | *"Seems as if the robot would need to be in that person’s life at the very early stages of cognitive deterioration so as to allow comfort, trust & acceptance."* (Yuan et al., 2022) |
|  |  |  | *“I would say earlier the better since it does the same thing at the same time everyday, that consistency is important for the person.”* (Arthanat and Begum, 2022) |
|  |  |  | *“The relationship has to be built from the early stage for comfort and dependability.”* (Arthanat and Begum, 2022) |
|  |  |  | *At the beginning “there would have to be another person there because I would be afraid to remain with it alone,” “to learn how to live together.”* (Sawik et al., 2023) |
| Design and Functionality |  | Individuals' perceptions of the robot’s physical design, usability, and effectiveness in performing tasks, which can influence their willingness to engage with the robot. | *“Should not be left on the floor where someone can trip over them.”* (Dosso et al., 2022) |
|  |  |  | *“They look artificial and futuristic…Plastic, alien looking,” “lifeless.”* (Dosso et al., 2022) |
|  |  |  | *“[F]rom what you say [the robot] can do I’d be happy with it but it’s going to get in the way. It’s going to have to use the other bedroom all the time...[The robot is] too tall and too wide.”* [Person E with MCI or ADRD] (Shin et al., 2022) |
|  |  |  | *"Developing a more human sounding voice is needed. The machine/computer voice would be extremely startling to a patient."* (Yuan et al., 2022) |
|  |  |  | *“I think it would be very important to have a New Zealand voice. Could be quite disorienting for people to have to interpret an accent, depending on the progression of the condition.”* [Provider] (Law et al., 2019) |
|  |  |  | *“I think he could speak more clearly. Some of the consonants I found difficult to understand.”* [Person with ADRD] (Law et al., 2019) |
|  |  |  | *"Not sure about size, not all patients have space for that size."* (Yuan et al., 2022) |
|  |  |  | *“Make sure that it can drive around chairs and plants; there will be clutter.” “Have you tried it with a pet? Is a dog going to chase it?” “It needs to drive on thick carpeting; what if carpets are not nailed down.”* (Arthanat and Begum, 2022) |
|  |  |  | *“I don’t have time for extra things that take time if it gets too complicated; Are we going to spend more time playing with it to try and make it run? Then we are caretaking it too.”* (Arthanat and Begum, 2022) |
|  |  |  | *“It moves slowly and I got impatient”* [Person with ADRD] (Raghunath et al., 2020) |
|  |  |  | *“Too slow to coming to help me with tasks…”* (Raghunath et al., 2020) |
|  |  |  | Care partner*: “The one about the companion, [she was like] “I don’t know about that.”* Person with ADRD*: It sounds a little creepy.* (Turner and Berridge, 2023) |
|  |  |  | *“I wonder about those big eyes; it might be a bit scary for an old person.”* [Provider] (Law et al., 2019) |
|  |  |  | *“Make it more human. It’s very casual because the person could put old clothing on top, that’s fine.”* [Person with ADRD] (Law et al., 2019) |
|  |  |  | *“I think I would make him [the robot] smaller. Especially because it moves around in small apartments and you might already have a walker too. You don't have a lot of space, right.”* (Van Assche et al., 2022) |
|  |  |  | *“When it comes to cleaning, [the robot] cleans only in the middle, not in the corners.” “It is not able because it’s a manual thing, to bend over and yet make an effort.”* (Sawik et al., 2023) |
| Caregiver and Care Receiver Misalignment |  | The extent to which both the caregiver’s and the care receiver’s needs, preferences, and expectations align regarding the robot's use, fostering cooperation and acceptance in caregiving situations. | *“. . .there may be some stuff that I need maybe more than you need, as far as my peace of mind, and I think that was really helpful to see it, to talk. You’re already really considerate and you want me to be out there, doing stuff and that might mean maybe some of the three other [technologies] that we’re not interested in, maybe that there’s space for you to think about that from that perspective.”* [Care partner] (Turner and Berridge, 2023) |
| Cost Concerns |  | Individuals’ worries about the financial burden associated with purchasing, maintaining, or repairing the robot | *“When I look at the cost over time, five to six thousand is not unreasonable at all.” “I’m paying 28$ an hour, five days a week, to my aide. If (the SAR) is not going to replace him, but I may be able to cut down on the hours.” “If you get an extra year or two at home, the cost is paid for.”* (Arthanat and Begum, 2022) |
|  |  |  | *“Hoping the government kicks in some money….;If there is possibility down the road to subsidies this technology.”* (Arthanat and Begum, 2022) |
|  |  |  | *“After it was done, I started realizing that we’re in the ozone, as far as no info on cost or installation. Costs of the basic electronics that are required, your iPhone, your pad, your whatever, whether it’s covered by Medicare and Medicaid or grants. No idea of the training.”* (Turner and Berridge, 2023) |
|  |  |  | *“I think it's going to be very expensive. That's why I think it's not going to be used quickly, the cost price should not be underestimated. […]. A robot also needs maintenance. […]. My idea is that, in the first place, it will be a luxury product for people who can afford it and still have enough capacity to use it. This would also be a department where you would have to pay more.”* (Van Assche et al., 2022) |
| ***Suggestions for Improving Robots for Independent Living*** | | | |
| Personalization and Adaptation |  | Enhancing the robot’s ability to adjust its features and interactions to fit the specific preferences, routines, and evolving needs of each user. | *“Well I think they would have to be tried to know. Yes, because there’s all grades of dementia as well isn’t there. And some would need more help than others.”* [Person with ADRD] (Law et al., 2019) |
|  |  |  |  |
|  | Personalized Training | Providing user-specific guidance and support to help individuals learn how to use the robot effectively based on their unique abilities and familiarity with technology. | *“If the university could offer a course or a workshop on this for caregivers…;” “demonstrate the technology to my family; someone needs to show me the protocols.”* (Arthanat and Begum, 2022) |
|  | Home Layout Considerations | Adjusting the robot’s design or functionality to navigate efficiently within different home environments, taking into account furniture arrangement, room sizes, and common obstacles. | *“We live in an old farmhouse, so there is nothing much we can do to create additional space; two level home is a concern and the robot will need information from places it can’t drive to; make sure there is WIFI connectivity across the house; my husband spends most of his day downstairs and the robot can drive to him in the living room and kitchen.”* (Arthanat and Begum, 2022) |
|  | Robot Function Personalization | Customizing the robot’s tasks and features to align with the individual user’s specific needs, preferences, and daily routines for enhanced relevance and usability. | *“[they would like the robot if] adding sensor accuracy, computing power and an easier or more responsive tablet.”* (Raghunath et al., 2020) |
|  |  |  | *“If they can put in the items they need, it would be more useful and customizable. There are different needs for different people.”* [Person with ADRD] (Law et al., 2019) |
| Enhancing Robot Features | Physical and Auditory Realism | Improving the robot’s physical appearance and sounds to feel more natural and lifelike, enhancing user comfort and emotional connection. | *“More friendly or familiar face on the screen is needed; I think it’s important that it is tall enough; Maybe use pre-recorded messages by family members.”* (Arthanat and Begum, 2022) |
|  | Friendlier Design and Pace of Movement | Enhancing the robot’s speed and responsiveness to better support timely assistance and align with users' pace in daily activities | *“I think it goes a little too fast for an older person.”* [Provider] (Law et al., 2019) |
|  |  |  | *“I like the questions it was asking and I think it was all quite manageable for someone with dementia.”* [Provider] (Law et al., 2019) |
|  |  |  |  |
| Advanced Functionalities | Voice Command and Emergency Features | Implementing intuitive and reliable voice control to allow users to interact with the robot hands-free and enhancing the robot’s ability to detect and respond quickly to emergencies | *“Would be great if they could provide a connection to emergency services if asked to do so.”* (Dosso et al., 2022) |
|  |  |  | *“I would [add voice activation] especially if someone has physical trouble, like if they’re [a person with disability]. That would be a big help.”* [Person B with MCI or ADRD] (Shin et al., 2022) |
|  |  |  | *“I don’t know whether my dad would find it as easy to use unless it is voice activated. Now [that] he has a voice control for the television [he] loves it*.” [Family caregiver B] (Shin et al., 2022) |
|  |  |  | *“Wish the robot can sense and alert if there is an abnormal heart rate or drop in blood pressure.”* (Arthanat and Begum, 2022) |
|  |  |  | *“Sound a siren or alarm first if the person tries to leave the house.”* (Arthanat and Begum, 2022) |
|  | Memory Assist Functions | Providing reminders and prompts to support users with memory tasks, such as medication schedules or appointments, helping them manage daily routines independently. | *“Also, remind people to wash, get cleaned up and ready for breakfast and this kind of thing. Sometimes for dementia people, they forget to do all these kinds of things.”* [Provider] (Law et al., 2019) |
|  |  |  | *“I think just having the reminder with the time can be quite helpful for someone that just can’t quite remember the checkpoints of the day, is not exactly sure about the passage of time, but can still do these tasks.”* [Provider] (Law et al., 2019) |
|  | Cognitive Games and Physical Activities | Offering engaging mental exercises to stimulate cognitive function and keep the user’s mind active as well as encouraging movement and physical exercise through interactive activities or reminders, supporting overall health and mobility. | *“Using the robot for activities would be nice. Like some kind of quiz games, where the robot can also keep track of the cognitive scores of the older adults.”* [Caregiver] (Van Assche et al., 2024) |

References

Arthanat, S., and Begum, M. (2022). *Effectiveness and adoption of a Smart home-based social assistive robot for care of individuals with Alzheimer’s Disease* (Grant Project Number: 1R01AG075892-01A1). Univeristy of New Hampshire.

Coşar, S., Fernandez-Carmona, M., Agrigoroaie, R., Pages, J., Ferland, F., Zhao, F., Yue, S., Bellotto, N., and Tapus, A. (2020). ENRICHME: Perception and Interaction of an Assistive Robot for the Elderly at Home. *International Journal of Social Robotics*, *12*(3), 779–805. https://doi.org/10.1007/s12369-019-00614-y

Dosso, J. A., Bandari, E., Malhotra, A., Guerra, G. K., Hoey, J., Michaud, F., Prescott, T. J., and Robillard, J. M. (2022). User perspectives on emotionally aligned social robots for older adults and persons living with dementia. *Journal of Rehabilitation and Assistive Technologies Engineering*, *9*, 20556683221108364. https://doi.org/10.1177/20556683221108364

Harris-Gersten, M. L., Davagnino, J. M., Alcorn, E. R., and Hastings, S. N. (2023). Usability and Acceptability of Social Robot Pets Among Community-Dwelling Veterans Living With Dementia and Their Caregivers. *American Journal of Alzheimer’s Disease & Other Dementias®*, *38*, 15333175231200973. https://doi.org/10.1177/15333175231200973

Law, M., Sutherland, C., Ahn, H. S., MacDonald, B. A., Peri, K., Johanson, D. L., Vajsakovic, D.-S., Kerse, N., and Broadbent, E. (2019). Developing assistive robots for people with mild cognitive impairment and mild dementia: A qualitative study with older adults and experts in aged care. *BMJ Open*, *9*(9), e031937. https://doi.org/10.1136/bmjopen-2019-031937

Portacolone, E., Halpern, J., Luxenberg, J., Harrison, K. L., and Covinsky, K. E. (2020). Ethical Issues Raised by the Introduction of Artificial Companions to Older Adults with Cognitive Impairment: A Call for Interdisciplinary Collaborations. *Journal of Alzheimer’s Disease: JAD*, *76*(2), 445–455. https://doi.org/10.3233/JAD-190952

Raghunath, N., Pereyda, C., Frow, J., Cook, D., and Schmitter-Edgecombe, M. (2020). A Robot Activity Support (RAS) system for persons with memory impairment: Comparing older and younger adults’ perceptions of the system. *Gerontechnology*, *19*(3), 1–11. https://doi.org/10.4017/gt.2020.19.003.07

Sawik, B., Tobis, S., Baum, E., Suwalska, A., Kropińska, S., Stachnik, K., Pérez-Bernabeu, E., Cildoz, M., Agustin, A., and Wieczorowska-Tobis, K. (2023). Robots for Elderly Care: Review, Multi-Criteria Optimization Model and Qualitative Case Study. *Healthcare (Basel, Switzerland)*, *11*(9), 1286. https://doi.org/10.3390/healthcare11091286

Shin, M. H., McLaren, J., Ramsey, A., Sullivan, J. L., and Moo, L. (2022). Improving a Mobile Telepresence Robot for People With Alzheimer Disease and Related Dementias: Semistructured Interviews With Stakeholders. *JMIR Aging*, *5*(2), e32322. https://doi.org/10.2196/32322

Turner, N. R., and Berridge, C. (2023). How I want technology used in my care: Learning from documented choices of people living with dementia using a dyadic decision making tool. *Informatics for Health and Social Care*, *48*(4), 387–401. https://doi.org/10.1080/17538157.2023.2252066

Van Assche, M., Petrovic, M., Cambier, D., Calders, P., Van Gelder, P., and Van De Velde, D. (2022). The perspectives of older adults with mild cognitive impairment and their caregivers on the use of socially assistive robots in healthcare: Exploring factors that influence attitude in a pre-implementation stage. *Disability and Rehabilitation: Assistive Technology*, *19*(1), 222–232. https://doi.org/10.1080/17483107.2022.2075477

Van Assche, M., Petrovic, M., Cambier, D., Calders, P., Van Gelder, P., Werner, F., and Van De Velde, D. (2024). Socially Assistive Robots in Aged Care: Expectations of Older Adults with MCI in Assisted Living Facilities and Their Caregivers. *International Journal of Social Robotics*, *16*(4), 687–698. https://doi.org/10.1007/s12369-024-01115-3

Van Orden, K. A., Bower, E., Beckler, T., Rowe, J., and Gillespie, S. (2022). The Use of Robotic Pets with Older Adults during the COVID-19 Pandemic. *Clinical Gerontologist*, *45*(1), 189–194. https://doi.org/10.1080/07317115.2021.1954122

Yuan, F., Anderson, J. G., Wyatt, T. H., Lopez, R. P., Crane, M., Montgomery, A., and Zhao, X. (2022). Assessing the Acceptability of a Humanoid Robot for Alzheimer’s Disease and Related Dementia Care Using an Online Survey. *International Journal of Social Robotics*, *14*(5), 1223–1237. https://doi.org/10.1007/s12369-021-00862-x
